# Dynamic behaviours of a flame as plasma in a strong electric field

**DOI:** 10.1038/s41598-019-50537-x

**Published:** 2019-11-01

**Authors:** Takao Fukuyama, Nodoka Mukai, Gaku Togawa

**Affiliations:** Faculty of Education, Nagasaki University, 1-14 Bunkyo-machi, Nagasaki, 852-8521 Japan

**Keywords:** Fluid dynamics, Plasma physics, Statistical physics, thermodynamics and nonlinear dynamics

## Abstract

Dynamic behaviours of a flame are experimentally examined via applying an electric potential difference between two parallel electrodes and placing a flame of ethanol between them. Given that a flame behaves as a weakly ionised plasma, the shape of the flame is affected by an external strong electric field. When a strong ac electric field is applied horizontally, i.e., transverse field, and as the applied ac voltage increases, the shape of the flame becomes flat and the width of the inner flame expands and saturates at a specific value. When a strong ac electric field is applied vertically, i.e., axial field, the frequency of the self-excited oscillation is affected by a specific value of the applied frequency. When the frequency of the applied ac voltage changes, temperature and light emission of flame are significantly affected by the applied ac voltage. Furthermore, when the strength of the horizontally applied electric field is further increased, a discharge occurs in the flame, and it is reshaped into an arc plasma. The current signals exhibit background troughs and sudden peaks in the form of spikes. The spectrophotometric curve includes the spectra of both the flames and arc plasma under the arc discharge.

## Introduction

Humans have used flames as sources of light and heat since the dawn of time, and flames play an important role in the development of civilization. In the early modern age, a famous book *The Chemical History of a Candle*^1^ by Michael Faraday expounded on the essential scientific features of flames, and it continues to be read throughout the world. Additionally, an understanding of flames is also important from a cultural perspective because flames are closely associated with our daily life and are essential to it. It is considered that the flame corresponds to the first form of plasma used by humans because a burning flame is an example of a weakly ionised plasma^2–5^. Human beings have used plasma in their daily life for a very long time, and studies were historically conducted on flames from the viewpoint of plasma^6,7^. A burning flame exhibits the characteristics of weakly ionised plasma because it is slightly ionised. The typical value of plasma density in the flame is around 10^8^ per 1 cm^2^. Therefore, the flame shape is affected by strong electric^3–5^ and magnetic^8,9^ fields. Plasma is produced in a diversified environment as a gas and also in liquid, solid, and supercritical states. To the best of the authors’ knowledge, studies do not examine the change from flame to plasma. Two studies considered flames as plasma and others searched for methods to control a flame via the methodologies of plasma physics. The studies are important given their applicability to the science of combustion such as microgravity^10^ and the removal of pollutants^11^. The aforementioned studies require analyses of the effects of electric fields on flames. The experiments throughout the study consider the properties of flame combustion of ethanol as a weakly ionised plasma in a strong electric field, response of flame’s shape to electric fields, observation and control of self-excited oscillation caused by thermal-diffusive instabilities, measurement of temperature and light emission of flame, and spectroscopies for the flame and arc discharge.

The present study reports on findings related to the dynamic behaviour of a flame of ethanol when it is subjected to an applied strong electric field. First, the method of changing the shape of a flame via subjecting it to a strong electric field is discussed. The relationship between the applied voltage and changes in the amount of flame are investigated for a few cases of frequencies of the applied voltage. Second, temperature and light emission of flame for the frequency of the applied voltage are examined in detail. Finally, the plasma produced by an arc discharge in a flame is explored in conjunction with the change from flame to arc plasma when the value of the applied voltage increases.

## Methods

### Flame for transverse electric field

**Figure 1:**
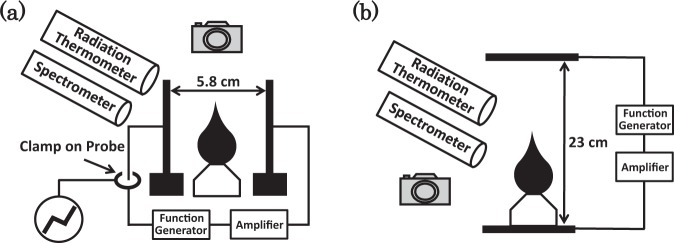
Schematic representation of the experimental configuration is shown, when a strong dc or ac electric field is applied (**a**) horizontally and (**b**) vertically. An ethanol lamp is placed between two stainless-steel electrodes that are arranged at a constant separation distance. A strong dc or ac electric field is applied between the two electrodes.

This study is performed via the experimental configuration shown in Fig. 1(a). A simple spirit (ethanol) lamp is placed between two stainless-steel electrodes that are arranged a certain distance apart. The distance between the two electrodes is fixed at 5.8 cm. The dimensions of each stainless-steel electrode are 18.0 cm × 7.0 cm. A high-speed camera (EX-F1, CASIO) with a lens (focus: 7.3~87.6 mm), digital oscilloscope (GDS-1072A-U, GWINSTEK), clamp on a probe (3276 and 3272, HIOKI), radiation thermometer (IR-AHS2, CHINO), and spectrometer (SEC2000 SPECTRA SYSTEM, ALS Co., Ltd) are used for the measurements. The high-speed camera is used in modes of 30 or 600 frames per second. High dc voltage is generated using a regulated dc power source (HV1.5–0.3, TAKASAGO). High ac voltage (which is used as rectangular voltage pulse in the study) is generated using a function generator (33220A, AGILENT), and the pulse (rectangular voltage pulse) is then amplified using a transformer (EF-4N, SHIMADZU) and an amplifier (4015, NF ELECTRONIC INSTRUMENTS). A strong dc or ac electric field is applied horizontally between the electrodes.

### Flame for axial electric field

We consider the dynamic behaviour of the flame with respect to axial electric field when the electric field is vertically applied. The study is performed using the experimental configuration shown in Fig. 1(b). Two stainless-steel electrodes are positioned in parallel with a certain vertical distance between them. The distance between the two electrodes is fixed at 23 cm. A strong ac electric field is applied vertically between the electrodes. A simple spirit (ethanol) lamp, high-speed camera, digital oscilloscope, radiation thermometer, and spectrometer are used for the measurements. High ac voltage (which corresponds to rectangular voltage pulse in the study) is generated using a function generator. Subsequently, the pulse (rectangular voltage pulse) is amplified via a transformer and an amplifier. The types of equipment are identical to that mentioned in the previous experiment.

## Discussions

### Shape of flame for transverse electric field

The space and time variations of the flame are evaluated when a strong electric field is applied between the electrodes. As is well known, the flame is drawn in the direction of the negative electrode when a strong dc electric field is applied. Photographs of the phenomenon are shown in refs^3,5,7^. The reasons for the phenomenon are as follows^3,5,7^. First, ions and electrons exist in a flame owing to vaporization of fuel, and micron-sized soot particles also exist in the background plasma (i.e., flame). Soot particles accumulate charge via electron attachment or via collisions in the flame. They lead to negative charge in the flame and are pulled towards the positive electrode when a dc field is applied. Second, positive ions are dominant in the flame because the electrons in the flame are lower owing to the movement of the negatively charged soot. Therefore, the flame is weakly positively charged and is drawn in the direction of the negative electrode.

**Figure 2:**
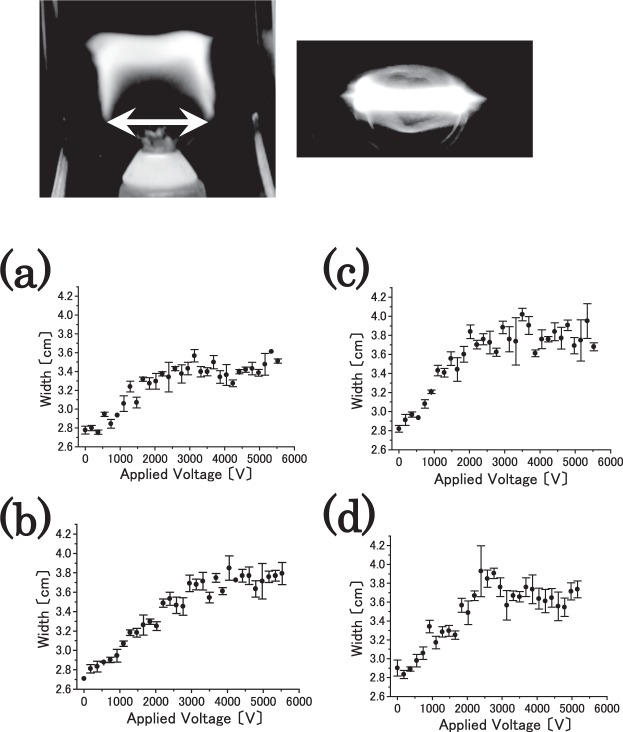
Change in the shape of the flame that occurs is shown when a strong ac electric field is applied. In upper trace, the photographs are obtained from the horizontal (left trace) and top (right trace) directions. In lower trace, relationship between the applied amount of ac voltage as rectangular voltage pulse and the width of the inner flame is shown. (**a**) 100 Hz, (**b**) 200 Hz, (**c**) 300 Hz, and (**d**) 400 Hz are shown.

As shown in upper traces of Fig. 2, the flame changes to a flat shape when a strong ac electric field is applied. A variable ac outlet and neon sign transformer (L704143G, LECIP) are used to produce a strong ac electric field in this case. The figure shows two photographs, namely one taken from the horizontal (upper left trace) and the other from the top (upper right trace) directions of the flame. This behaviour is explained as follows^3^. First, the flame is drawn to the negative electrode because it is weakly positively charged as previously mentioned. Second, the cathode switched at a high speed because the electric field oscillated with a period of $\frac{1}{60}$ s. The flame could not follow the oscillation, and thus the flame is flattened.

The response of the flame to the applied ac voltage is quantitatively examined. A simple flame (for e.g., a candle or spirit lamp) exhibits a structure that is divided into outer and inner regions^1,3^. The flame in the inner region is covered by that in the outer region. With respect to the flame in the outer region, an oxidation reaction actively progresses with the emission of light, and a strong thermal release is observed because the flame is in full contact with the oxygen in the atmosphere. The outer flame is extremely susceptible to external perturbations, such as air flow and electromagnetic fields, and therefore moves intensely. Therefore, it is difficult to quantitatively measure the shape of the outer flame. Conversely, the oxidation reaction in the inner flame is slower because the amount of oxygen is insufficient because it is mostly consumed in the outer flame. Chemical reactions including oxidation in the inner flame do not occur as often as those in the outer flame. The electric field does not significantly affect the inner flame given the lack of combustion, plasma, and soot. Therefore, a net positive charge is absent, and the inner flame barely moves. Given the aforementioned reasons, it is possible to quantitatively measure the shape of the inner flame.

### Relationship between the magnitude of the applied voltage and width of the inner flame

We examine the response of the flame to the horizontally applied ac voltage quantitatively. The region denoted by the arrow in photograph in Fig. 2 is measured because it corresponds to the maximum width in the inner flame. Specifically, the inner flame symmetrically appears between the parallel stainless-steel electrodes. The flame spread is measured by averaging over multiple recorded images via a high-speed camera. It is noted here that the symmetric appearance of the inner flame collapses when a strong dc electric field is applied, and it is not possible to measure the width of the inner flame because the flame is drawn in the direction of the negative electrode.

Figure 2 shows the relationship between the applied amount of ac voltage (which corresponds to rectangular voltage pulse in the experiment) and the width of the inner flame. Frequency of ac voltage (which is rectangular voltage pulse in this case) is varied as a control parameter. As shown in Fig. 2, the variations in the ac voltage frequencies include (a) 100 Hz, (b) 200 Hz, (c) 300 Hz, and (d) 400 Hz. In the figures, the error bars represent the standard deviation, which is calculated based on five measurements for each parameter. When the applied ac voltage increases, the width of the inner flame saturates above a certain value. The reason as to why the inner flame saturated is as follows: The width of inner flame spreads with respect to increases in the strength of the applied voltage. However, even if a stronger electric field is applied, it is not likely that the system exceeds the threshold to cause breakdown of atmospheric air as insulation. Therefore, the width of the inner flame saturates above a certain value.

### Self-excited oscillation of flame in the vertical direction with respect to axial electric field

**Figure 3:**
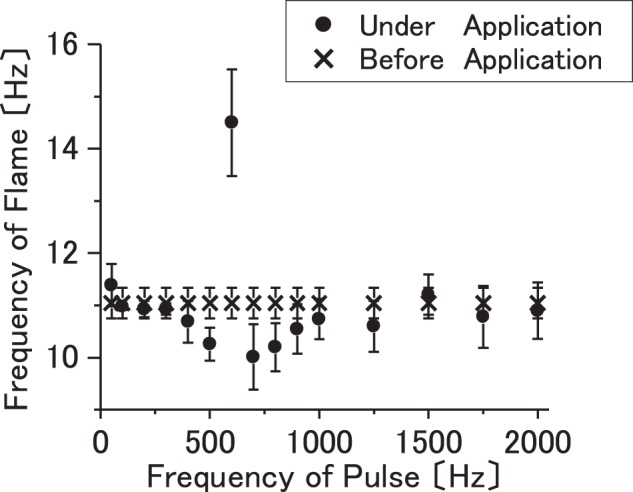
Frequency of the self-excited oscillation of the flame with respect to the frequency of vertically applied electric field are shown. Amplitude of applied electric field is fixed at 5152 V.

The flame exhibits a self-excited oscillation in the vertical direction. It is inferred that the self-excited oscillation is caused by the effects of the evaporation of the fuel and buoyancy effects, i.e., the thermal–diffusive instabilities as observed in the flickering of any other diffusion flame such as a candle^12^. In the experimental procedure, the ac voltage is vertically applied as an external force that affects the self-excited oscillation of the flame. The frequency of the self-excited oscillation of the flame approximately corresponds to 11 Hz prior to the application of the external force. The frequency of the self-excited oscillation of the flame typically corresponds to approximately 10 Hz in several cases of the experiments^13–15^. Here, the frequency of the self-excited oscillation is counted via playing a high-speed camera recording in slow motion. Subsequently, the ac electric field is applied between the electrodes as an external force and is continuously changed. Amplitude of the applied electric field is fixed at 5152 V. Figure 3 shows the experimental results, i.e., the frequency of the self-excited oscillation of the flame with respect to the frequency of the vertically applied electric field. In the figure, the error bars represent the standard deviation, which is calculated based on seven measurements for each parameter. When the applied ac frequency is changed, the frequencies of the self-excited oscillation are not significantly affected by the applied electric field with the exception of the case corresponding to 600 Hz. In the case when the applied electric field corresponds to 600 Hz, the frequency of the self-exited oscillation is significantly affected by the applied electric field, and it exceeds 14 Hz. Therefore, the results indicate that the self-excited oscillation of the flame is significantly affected from a specific frequency of an external electric field.

### Change of temperature and light emission of flame with respect to axial electric field

**Figure 4:**
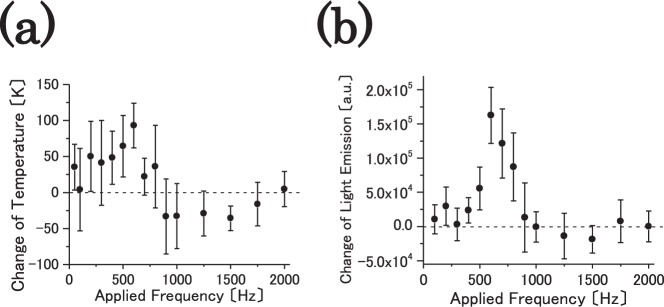
(**a**) Change in flame temperature and (**b**) change in flame light emission for frequency of applied ac voltage as rectangular voltage pulse are shown. Amplitude of applied electric field is fixed at 5152 V.

Subsequently, Fig. 4 shows the changes in (a) flame temperature and (b) flame light emission with respect to the frequency of the applied ac voltage as pulse (rectangular voltage pulse). The amplitude of the applied electric field is fixed at 5152 V. As a consequence, it is considered that flame temperature and flame light are significantly affected by the applied ac voltage. The flame temperature and light emission evidently increase or decrease at 900 Hz (i.e., the frequency of applied ac electric field) as the threshold. Furthermore, the flame temperature increases by approximately 95 K at 600 Hz. Additionally, it decreases by approximately 35 K at 1500 Hz as shown in Fig. 4(a). The average value of flame temperature increases from 941 K to 1034 K in case the value of applied frequency is 600 Hz, and it decreases from 989 K to 953 K in case of 1500 Hz. The change in the flame light emission exhibits a relationship with the change in flame temperature. Hence, the possibility of controlling flame temperature via the frequency of the applied ac electric field is shown.

Furthermore, we consider the relationship between change in flame temperature and burning speed which relates to the amount of ethanol that is consumed. Specifically, we focus on the case wherein the frequency of the applied electric field corresponds to 600 Hz, which in turn mainly increases the flame temperature. Furthermore, we focus on the case wherein the frequency is 1500 Hz, which in turn primarily decreases the flame temperature. Results of the burning speeds including the standard deviation are as follows: prior to the application of the external electric field; 0.819 ± 0.011 g per minute, 600 Hz 5152 V; 0.803 ± 0.018 g per minute, 600 Hz 7360 V; 0.775 ± 0.018 g per minute, 1500 Hz 5152 V; 0.841 ± 0.010 g per minute.

In a practical context with respect to the aforementioned results, burning speed naturally also increases based on increases in flame temperature. However, contrary to our expectations, burning speed decreases with increases in flame temperature, and vice versa (i.e., it increases with decreases in flame temperature). To further understand the reasons, measurements of ion density and ionization degree in flame via the probe and spectroscopic method should be examined in a future study.

### Flame and arc studies

When the ac electric field (sine wave, 60 Hz) is applied horizontally as shown in Fig. 1(a) and becomes extremely strong, electric current flows in the flame and an arc discharge occurs. The flame is ionised by the electric current and becomes an arc plasma because the flame exhibits higher conductive properties than atmospheric air. It is expected that an arc plasma does not occur without a flame when a strong voltage is applied between the electrodes because the two electrodes are held at a sufficient distance from each other. It should be noted that for the flat plate, the sparking voltage at which an arc forms in air is estimated based on an extant study^16^. The estimated value for the plate separation in the experiment approximately corresponds to 184 kV. However, the sparking voltage can become significantly lower than the estimated value owing to the conductivity of the flame. When the ac voltage applied to the flame is continuously increased, the shape of the flame begins to change.

**Figure 5:**
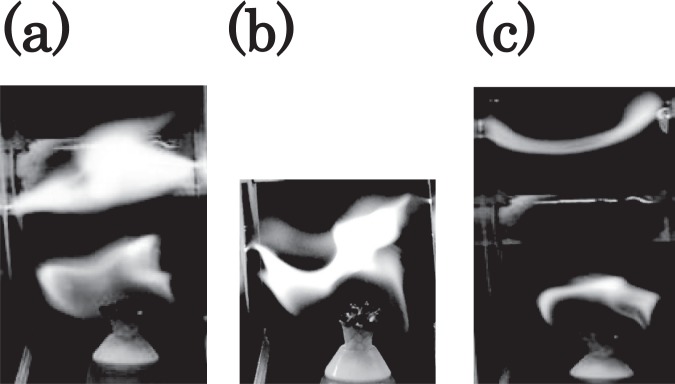
Change in the flame state for an extremely strong electric field is shown: (**a**) appearance of arc plasma when an ac voltage corresponding to 16,500 V is applied, (**b**) mixing state of the flame and arc plasma when an ac voltage corresponding to 16,500 V is applied, and (**c**) state when the filament of arc plasma began to run from the bottom to top between the electrodes when an ac voltage corresponding to 18,090 V is applied.

The flame is drawn towards both electrodes when the electric ac field is applied, and the electric current flows in the flame. The resulting arc plasma appears at approximately 16,500 V as shown in Fig. 5 (a). The photographs in Fig. 5 correspond to the typical instantaneous images that are taken via a high-speed camera at 600 frames per second. The mixing state of the flame and arc plasma are shown in Fig. 5(b). In Fig. 5(a,b), an ac voltage of 16,500 V is applied. With respect to a stronger voltage, a filament of the arc plasma begins to run from the bottom to top between the electrodes as shown in Fig. 5(c). Subsequently, the flame component decreases. The system changes to blue-white emission, and this is a characteristic of an arc plasma. At this point, an ac voltage of 18,090 V is applied.

**Figure 6:**
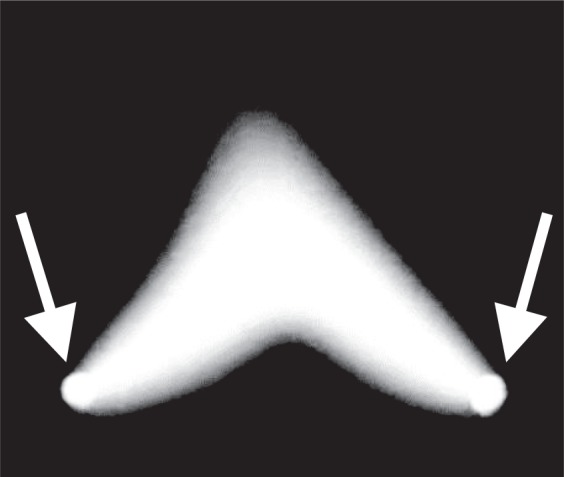
Arc plasma produced between the stainless-steel electrodes with a nail shape under normal atmospheric air is shown.

Figure 6 shows a typical image depicting the arc plasma produced between the stainless-steel electrodes with a nail shape under normal atmospheric air. An arc bright spot is observed in the area at the tip of the nail corresponding to the root of the arc discharge. Their spots are indicated by the arrows in Fig. 6. The arc plasma assumes a typical upward convex shape as shown in Fig. 6. The cause of the phenomenon is similar to that of the behaviour of the central part of an arc plasma filament in the pathway of discharge that is lifted up because of a vertical updraft of heat. Conversely, the arc plasma shown in Fig. 5(c) exhibits an upward concave shape, and this differs from the typical one as shown in Fig. 6. The arc plasma filament convects upward and maintains a stable shape. To the best of the authors’ knowledge, previous studies do not explain the phenomenon. We attribute the phenomenon to the following. First, the arc plasma filament freely runs from the bottom to top between the electrodes because both ends of the discharge are not fixed. Second, both sides of the arc plasma filament move quickly upward, and the filament takes on an upward concave shape.

**Figure 7:**
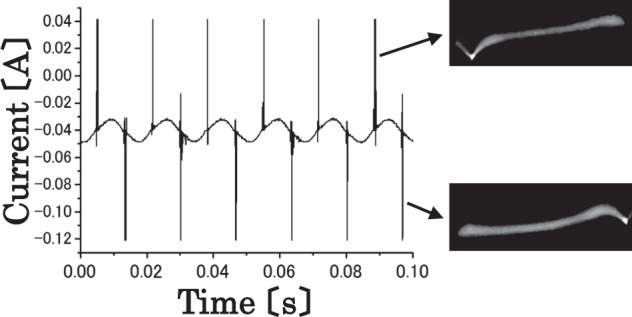
Time series of the discharge current measured via using a clamp on the probe is shown when an ac voltage of 16,500 V is applied and arc discharge occurs. Photographs of the discharge are shown.

The time series of the discharge current is measured via a clamp on the probe when the arc discharge occurs. As expected, the current signal is not observed when the discharge does not occur. When the discharge occurs, the current signals exhibit background troughs and sudden peaks in the form of spikes as shown in Fig. 7. Specifically, an ac voltage of 16,500 V is applied. The current does not flow continuously, and the discharge repeats intermittently owing to variations in the conductivity of the arc with sudden peaks in the form of spikes of approximately 120 Hz. Photographs of the discharge are also shown in Fig. 7. Arc bright spots that are observed on both electrodes are highlighted. First, a strong luminous point appears on the left electrode prior to appearing on the right electrode after $\frac{1}{120}$ s. Therefore, the arc bright spot on both electrodes becomes strongly luminous in alternate shifts. The strong luminosity corresponds to the peaks of the discharge current because the intervals of the strong luminosity are identical to that of the peaks.

**Figure 8:**
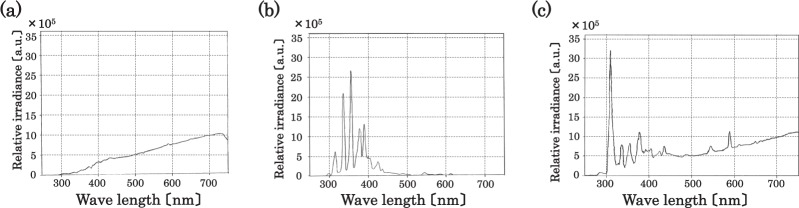
Spectrophotometric curve obtained using a spectrometer is shown. The spectra of the (**a**) flame, (**b**) arc discharge, and (**c**) flame and arc plasma under the arc discharge, are shown.

The observed spectrophotometric curve using a spectrometer is shown in Fig. 8. Figure 8(a–c) show the spectra of the flame, arc discharge, and flame and arc plasma under the arc discharge, respectively. The spectrophotometric curve in the range 250 to 750 nm is measurable owing to the technical limitations of the diagnostic for wavelength. In Fig. 8(a,c), a broad curve and infrared radiation caused by the flame is observed. Several peaks are shown in Fig. 8(b,c) including the ultraviolet radiation caused by the arc discharge corresponding to the spectra of the ionised atmosphere (e.g., nitrogen). The emissions of light from the flame and ionised atmosphere mix with each other under the arc discharge as shown in Fig. 8(c). The result indicates that the flame and arc discharge simultaneously coexist. Based on the results for an arc discharge in a flame, the following conclusions are drawn. First, a discharge in a flame can occur at a significantly lower voltage than that of a discharge in the atmosphere because a flame exhibits higher conductive properties^17–20^ than the atmosphere. Second, a discharge in a flame between parallel stainless-steel electrodes exhibits unique shape characteristics when compared to a typical arc discharge, and the arc bright spot forms between electrodes exhibits a nail shape under normal atmospheric air. A series of experiments is conducted on a simple flame (ethanol lamp). It is anticipated that the simplicity of our experimental setup is potentially relevant to other systems, i.e., different flames, such as a Bunsen burner.

## Conclusion

In conclusion, the findings obtained in the investigation are summarized as follows. Experiments are conducted to investigate the dynamic behaviour of simple flames in strong electric fields. The flame adopts a flat shape when a strong ac electric field is applied horizontally. The width of the inner flame expands and saturates when the applied amount of ac voltage increases. The frequency of the self-excited oscillation is affected by a specific value of the applied frequency when a strong ac electric field is applied vertically. Temperature and light emission of flame are significantly affected by the applied ac voltage. An arc discharge occurs in the flame when the applied transverse electric ac field increases. The flame is ionised by the electric current that flows into the flame and becomes an arc plasma. The filament of the arc plasma in the flame produces an upward concave shape, and this differs from the shape of typical arc plasma. When an arc discharge is observed in the flame, the current signals exhibit background troughs and sudden peaks in the form of spikes of 120 Hz. The spectrophotometric curve includes mixed emissions of light from the flame and ionised atmosphere under the arc discharge. The study demonstrates that an electric field can be used to control the motion and temperature of a flame.

## Data Availability

The datasets generated during our study are available from the corresponding author on reasonable request.
